# The double-stranded break-forming activity of plant SPO11s and a novel rice SPO11 revealed by a *Drosophila *bioassay

**DOI:** 10.1186/1471-2199-13-1

**Published:** 2012-01-16

**Authors:** Yoshinori Shingu, Takeshi Tokai, Yasuo Agawa, Kentaro Toyota, Selina Ahamed, Makiko Kawagishi-Kobayashi, Akira Komatsu, Tsutomu Mikawa, Masa-Toshi Yamamoto, Kyo Wakasa, Takehiko Shibata, Kohji Kusano

**Affiliations:** 1 Cellular & Molecular Biology Laboratory, RIKEN Advanced Science Institute, 2-1 Hirosawa, Wako, Saitama 351-0198, Japan; 2 Department of Supramolecular Biology, Graduate School of Nanobioscience, Yokohama City University, Tsurumi-ku, Yokohama, Kanagawa 230-0045, Japan; 3 Department of Agriculture, Tokyo University of Agriculture, Atsugi, Kanagawa 243-0034, Japan; 4 Center for Genetic Resource Education & Development, Kyoto Institute of Technology, Saga-Ippongi-cho, Ukyo-ku, Kyoto 616-8354, Japan; 5 National Institute of Crop Science, 2-1-8 Kannondai, Tsukuba, Ibaraki 305-8518, Japan

## Abstract

**Background:**

SPO11 is a key protein for promoting meiotic recombination, by generating chromatin locus- and timing-specific DNA double-strand breaks (DSBs). The DSB activity of SPO11 was shown by genetic analyses, but whether SPO11 exerts DSB-forming activity by itself is still an unanswered question. DSB formation by SPO11 has not been detected by biochemical means, probably because of a lack of proper protein-folding, posttranslational modifications, and/or specific SPO11-interacting proteins required for this activity. In addition, plants have multiple SPO11-homologues.

**Results:**

To determine whether SPO11 can cleave DNA by itself, and to identify which plant SPO11 homologue cleaves DNA, we developed a *Drosophila *bioassay system that detects the DSB signals generated by a plant SPO11 homologue expressed ectopically. We cytologically and genetically demonstrated the DSB activities of *Arabidopsis *AtSPO11-1 and AtSPO11-2, which are required for meiosis, in the absence of other plant proteins. Using this bioassay, we further found that a novel SPO11-homologue, OsSPO11D, which has no counterpart in *Arabidopsis*, displays prominent DSB-forming activity. Quantitative analyses of the rice SPO11 transcripts revealed the specific increase in OsSPO11D mRNA in the anthers containing meiotic pollen mother cells.

**Conclusions:**

The *Drosophila *bioassay system successfully demonstrated that some plant SPO11 orthologues have intrinsic DSB activities. Furthermore, we identified a novel SPO11 homologue, OsSPO11D, with robust DSB activity and a possible meiotic function.

## Background

Homologous genetic recombination plays critical roles in meiosis, for genetic diversification and precise disjunction of homologous chromosomes. In the budding yeast *Saccharomyces cerevisiae*, meiotic recombination is initiated by chromatin locus- and timing-specific DNA double-strand breaks [1-3], which require the function of the SPO11 protein [4,5]. *Saccharomyces cerevisiae *SPO11 shares amino acid sequence homology with subunit A of the type II DNA topoisomerase (topoisomerase VI) from the archaeon *Sulfolobus shibatae *[5]. Type II topoisomerases generate double-strand breaks (DSBs) at specified sequences in the cleavage complex, an intermediate for topological DNA reactions, composed of the covalent complex of a topoisomerase subunit and the terminus of each DSB strand. As in the cleavage complex, SPO11 covalently attaches to the 5' termini of a nascent meiotic DSB. SPO11 is then removed from the DNA by the endonuclease activity of Mre11 [6-9], and by the functions of the SAE2/CPT1 [10,11] and RAD50 proteins [10], but not by Nbs1, the third component of the MRN complex [8,9].

SPO11 is ubiquitous in eukaryotes, including *S. cerevisiae *[4], the fission yeast *Schizosaccharomyces pombe *(Rec12) [12], the fruit-fly *Drosophila melanogaster *(Mei-W68) [13], the nematode *Caenorhabditis elegans *(T05E11.4) [14], the basidiomycete *Coprinus cinereus *[15] and mammals [16], and each has a single gene encoding a SPO11-orthologue. Thus, it has been assumed that SPO11 is the essential protein to introduce DSBs for the initiation of meiotic recombination in eukaryotes [see [17]].

In contrast to these organisms, searches for homologues of SPO11 or TOP6A (subunit A of topoisomerase VI) in genome databases revealed that plants have multiple SPO11-homologues: *Arabidopsis thaliana *has AtSPO11-1, AtSPO11-2 and AtSPO11-3 [18,19], and their homologues in *indica *rice (*Oryza sativa *L.) are OsTOP6A1 (OsSPO11A), OsTOP6A2 (OsSPO11B) and OsTOP6A3 (OsSPO11C), respectively [20]. Genetic studies revealed that efficient meiotic recombination in *Arabidopsis *depends on both the *AtSPO11-1 *[21] and *AtSPO11-2 *[22] gene products, which are involved in the induction of DSBs and the initiation of meiosis. In contrast, *AtSPO11-3 *plays a major role during somatic cell development, but not in meiosis and meiotic recombination. The loss of the *AtSPO11-3 *function results in abnormal endoreduplication, an extreme dwarf phenotype and deficient cell proliferation [23-25]. AtSPO11-2 and AtSPO11-3 interact with AtTOP6B (subunit B of the archaeal topoisomerase VI), as shown by two-hybrid studies [19], suggesting that they function as DNA topoisomerases. In rice, *OsTOP6A1 *(*OsSPO11A*) is required in rice meiosis [26]. As with *AtSPO11-3*, overexpression studies in *Arabidopsis *suggested the functions of *OsTOP6A3 *(*OsSPO11C*) and *OsTOP6B *in stress adaptation in mitosis [20].

Genetic studies indicated that SPO11s play critical roles in DSB formation, for the initiation of meiotic recombination. In *S. cerevisiae*, meiotic DSB formation for meiotic recombination initiation requires the products of at least nine genes (*i.e*., *REC102, SKI8, REC104, REC114, MEI4, MER2, MRE11, RAD50 *and *NBS1*/*XRS2*), in addition to that of the *SPO11 *gene [See [17] for review, [27]]. However, many of these proteins are not conserved in other organisms. Although MRE11, RAD50 and XRS2 are respectively conserved in mammals as Mre11, Rad50 and Nbs1, and in *Arabidopsis *as AtMRE11, AtRAD50 and AtNBS1, their corresponding proteins are not required for meiotic DSB formation [28,29]. Instead, for meiotic DSB formation, *Arabidopsis *requires PRD1, which appears to be an orthologue of mammalian Mei1 [30], while *D. melanogaster *requires Mei-P22 [31], which has no homologue in other eukaryotes. These observations suggested that higher eukaryotes and yeasts differ in their activation control of meiotic DSB formation and in their protein requirements for SPO11 to express its DSB-forming activity.

Genetic studies demonstrated the activity of SPO11 to generate meiotic DSBs, but the biochemical function of SPO11 is essentially unknown. We previously isolated a soluble form of AtSPO11-1, with a DNA binding activity that functions in meiosis [32], and other plant SPO11s (Y. S. unpublished observations). However, the DSB-forming activities of AtSPO11 alone, with or without an attached tag, and of the complex of AtSPO11 with a protein (PRD1) required for SPO11-dependent DSB formation have not been detected *in vitro *[32]. The inability to detect the DSB-forming activities of SPO11 *in vitro *may simply be due to the *in vitro *conditions chosen for the tests, the improper folding and absence of eukaryote-specific posttranslational modifications of the protein expressed within bacterial cells, the unsuccessful multimer formation, or the absence of required SPO11-interacting proteins.

To overcome these difficulties in determining whether SPO11 by itself has DSB-forming activity and which plant SPO11 candidates function in DSB formation for meiotic recombination initiation, we tried to develop an *in situ *assay for DSBs, by expressing a plant SPO11 in eukaryotic cells. We chose the fruit-fly, *Drosophila melanogaster*, in which the expressed proteins would be folded and modified under the *in vivo *conditions of eukaryotic cells and the chromatin is loosened during meiosis to allow the access of SPO11 to the canonical DSB sites in chromosomal DNA [33-35]. In addition, many well-established cytological and genetic techniques are available for *Drosophila*. The DSBs generated by the plant SPO11 were detected by *in situ *immunostaining, using an antibody that recognizes *Drosophila *phosphorylated histone H2Av (γ-H2Av). H2Av is the sole *Drosophila *H2A variant and the functional homolog of human H2AX. The phosphorylated form of H2Av accumulates at DSB sites *in Drosophila *[36-39], as in mammals [40,41]. Genetic analyses of the consequences of the ectopic expression of plant SPO11s in *Drosophila *would provide further support for their DSB-forming activities.

Here, we report quantitative analyses of the DSB-forming activities of plant SPO11s in the absence of plant SPO11-interacting proteins in *Drosophila *oocytes, as a bioassay system. The DSB formation in the oocytes was further confirmed by measuring the meiotic loss of the X chromosome, which was expected as the result of abortive DSB formation in meiosis, in the absence of authentic SPO11. In addition, using these assays, we tested three candidates of meiotic SPO11 in rice, and identified a novel SPO11 homologue, OsSPO11D, which possesses DSB-forming activity and is expressed specifically in meiotic tissue.

## Results

### Detection of DSB signals induced by plant SPO11s expressed in *Drosophila *oocytes

Since a biochemical assay was not available, in order to address the questions of whether SPO11 has DNA cleavage activity by itself and which rice SPO11 candidates function in DSB formation for rice meiotic recombination, at first we tested the rice SPO11 homologues, OsSPO11A OsSPO11B and OsSPO11C, which phylogenetically correlate with AtSPO11-1, AtSPO11-2 and AtSPO11-3 (which lacks meiotic function), respectively (see later section), and the novel fourth homologue, along with the *Arabidopsis *SPO11 homologues, AtSPO11-1 and AtSPO11-2, for the heterospecific complementation of the reduced fertility of the *Arabidopsis spo11-1 *mutant. Only AtSPO11-1 complemented the deficiency of the mutant. These negative results and the advantages of the *Drosophila *systems as described prompted us to test *Drosophila *meiotic recombination mutants, to address the above questions.

A cytological technique to detect DSBs is available in *Drosophila*, by the use of an antibody against a phosphorylated human histone H2A variant, γ-H2AX, in which serine 139 is phosphorylated upon DSB formation [40,42,43]. The anti-human γ-H2AX antibody recognizes γ-H2Av, the phosphorylated *Drosophila *histone H2A variant, H2Av, which is phosphorylated after DSB formation induced either exogenously or during meiosis [36-38]. This technique revealed that in wild type *Drosophila*, DmSPO11 (Mei-W68)-dependent DSBs appeared transiently in region 2b of the germarium, in which the γ-H2Av signals overlap with the chromosomal DNA signals and disappear in region 3 (stage 1) [see ref. [37] about regions and stages]. In postmeiotic nuclei of stage 3 or later egg chambers, the chromosomes are condensed to form a compact karyosome.

In yeast, unrepaired Spo11-dependent meiotic DSBs reportedly accumulated in DSB-repair defective mutants, such as *rad50S*, and the DSB-repair defective mutants facilitated the sensitive mapping and reliable quantification of the SPO11-dependent meiotic DSB formation [3,44,45]. In DSB-repair deficient mutant flies (*SPO11*-proficient), such as *mus301, spn-A *(encoding a RAD51 homologue), *spn-B *(encoding another RAD51 homologue) and *okr *(encoding a RAD54 homologue), unrepaired meiotic DSBs accumulate and persist in the oocyte nuclei of the stages beyond the germarium region 2b of ovaries [37,38], and cause defective karyosome morphology (partially compact karyosome) in stage 3 or later egg chambers [46]. Thus, this DSB-repair defective phenotype provides a sensitive and reliable measure of SPO11-dependent meiotic DSBs.

**Figure 1:**
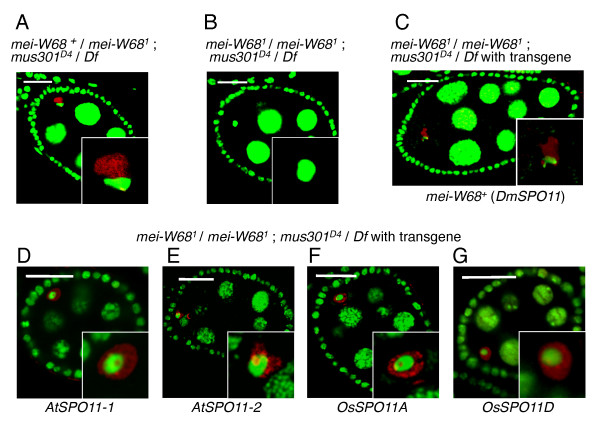
**Confocal images of DSB signals generated by plant *SPO11 *expression in the *mei-W68 mus301 *double mutant background (*mei-W68*^*1*^/*mei-W68*^*1*^; *mus301*^*D4*^/*Df(3L)66C-G28*)**. The DNA is green. The γ-H2Av is red, and shows the DSB signals. When the two signals overlap, the signal is yellow. Each inset shows an enlarged view of the oocyte nucleus. All images are single confocal sections. Each scale bar shows 20 μm. **(A) **An egg chamber of *mei-W68^+ ^*/*mei-W68*^*1*^; *mus301*^*D4*^/*Df(3L)66C-G28*. **(B) **An egg chamber of *mei-W68*^*1*^/*mei-W68*^*1*^; *mus301*^*D4*^/*Df(3L)66C-G28*. The frequencies of the oocyte nuclei with DSB signals for the total oocyte nuclei of stage 2-8 egg chambers were 100% (344/345, 3 ovaries) in the *mei-W68*^*1 *^heterozygote, and 7.4% (16/220, 2 ovaries) in the *mei-W68*^*1 *^homozygote (see Figure 2A). The frequencies of the oocyte nuclei with a karyosome morphological defect (Figure 1) for the total oocyte nuclei of stage 3-8 egg chambers were 91% (254/278, 3 ovaries) in the *mei-W68 *heterozygote, and 1.3% (2/177, 2 ovaries) in the *mei-W68 *homozygote (see Figure 2B). **(C) **An egg chamber of *mei-W68*^*1 *^/*mei-W68*^*1*^; *mus301*^*D4 *^/*Df(3L)66C-G28 *with transgene *P{hsp83-mei-W68^+^cDNA, M53-3} *(indicated as *Vector name{gene, insertion #}*). The frequency of the oocyte nuclei with DSB signals for the total oocyte nuclei of stage 2-8 egg chambers was 100% (180/180, 2 ovaries) in the *mei-W68 *homozygote with *P{hsp83-mei-W68^+^cDNA, M53-3}*, similar to the *mei-W68*^*1 *^heterozygote. The frequency of the oocyte nuclei with defective karyosome morphology for the total oocyte nuclei of stage 3-8 egg chambers was 70% (93/132) in the *mei-W68 *homozygote with *P{hsp83-mei-W68^+^cDNA, M53-3}*, similar to the *mei-W68*^*1 *^heterozygote. The karyosomes in the insets of panels A and C (SPO11 positive oocyte with DSB repair deficiency, as positive controls) have defective morphology (non-disc shape), while the karyosome in the inset of panel B (DSB-repair defective oocyte without functional SPO11 as negative controls) has normal morphology (disc shape). **(D-G) **Egg chambers of *mei-W68*^*1 *^/*mei-W68*^*1*^; *mus301*^*D4 *^/*Df(3L)66C-G28 *with transgene: *P{hsp83-AtSPO11-1 cDNA, A5-1} *(D); *P{hsp83-AtSPO11-2 cDNA, 2M3-1} *(E); *P{hsp83-OsSPO11A cDNA, 3M2-3} *(F); *P{hsp83-OsSPO11D cDNA, F2-1} *(G). Quantitative data with respect to the DSB signals and the defective karyosome morphology in the oocyte nuclei of these transgenic flies are shown in Figure 2.

In our positive control experiment, *mei-W68*^*1 *^heterozygous (*DmSPO11*-proficient phenotype) and *mus301 *hemizygous (*mus301-*defective phenotype) flies showed the extensive accumulation of γ-H2Av signals in almost all oocyte nuclei at stage 2-8 egg chambers, and defective karyosome morphology in most oocyte nuclei at stage 3-8 egg chambers (100% and 91%, respectively, Figure 1A). The γ-H2Av signals in the oocyte nuclei were red outside the partially compact karyosome, and some yellow signals (indicating overlap with DNA) were present in a tiny area within the stained DNA (green) area (Figure 1A and 1C). This slight overlap of the DSB signals and the major DNA signals was previously reported: while the normal karyosome in DNA repair-proficient oocytes is condensed in stage 3 or later and visualized by nuclear staining (green), in the oocyte nuclei of the DSB repair-deficient mutant *spn-A*, a *rad51 *paralogue, the unrepaired DNA is dispersed and not visible by nuclear staining, except for a tiny overlapping region (yellow). However, the unrepaired DSBs were recognized by the anti-γ-H2AX antibody (red) as γ-H2Av foci outside the partially condensed karyosome, where the unrepaired chromosomal DNA is dispersed in the nuclei [37].

**Figure 2:**
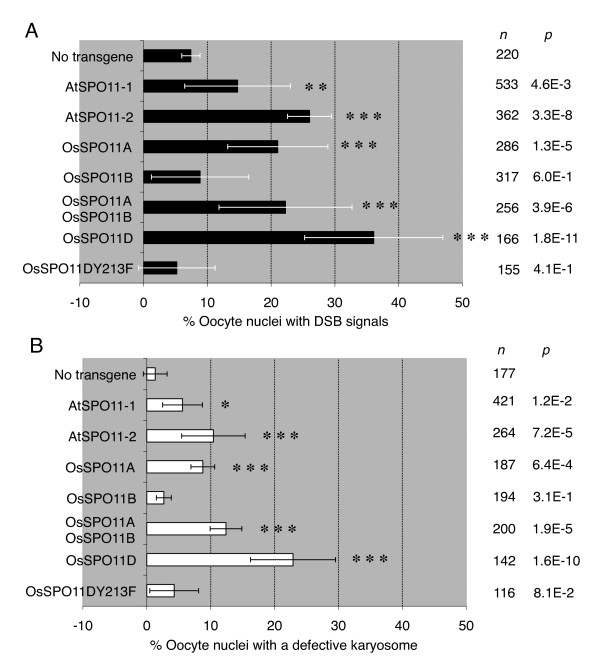
**DSB formation by plant *SPO11 *expression in the *mei-W68 mus301 *double mutant background (*mei-W68*^*1 *^/*mei-W68*^*1*^; *mus301*^*D4 *^/*Df(3L)66C-G28*)**. **(A) **Oocyte nuclei with DSB signals, which were detected by staining γ-H2Av, were scored in stage 2-8 egg chambers. The number of oocyte nuclei with DSB signals was divided by the total number of oocyte nuclei observed. **(B) **Oocyte nuclei with defective karyosome morphology, which was observed as a non-disc shape, were scored in stage 3-8 egg chambers. The number of oocyte nuclei with defective karyosome morphology was divided by the total number of oocyte nuclei observed. The values of the positive control (bearing the *hsp83-mei-W68*^*+ *^transgene) for panels A and B were described in the legend to Figure 1C. Transgene (*Vector name{gene, insertion #}*) [Number of ovaries used for observations]: *P{hsp83-AtSPO11-1 cDNA, A5} *[7]; *P{hsp83-AtSPO11-2 cDNA, M3-1} *[3]; *P{hsp83-OsSPO11A cDNA, 3M2-3} *[3]; *P{hsp83-OsSPO11B cDNA, M6-2} *[2] and *P{hsp83-OsSPO11B cDNA, M7-1} *[1]; *P{hsp83-OsSPO11A cDNA, 3M2-3} with P{hsp83-OsSPO11B cDNA, F3-2} *[2]; *P{hsp83-OsSPO11D cDNA, F2-1} *[2]; *P{hsp83-OsSPO11D^Y213F^, M33-F1} *[2] and *P{hsp83-OsSPO11D^Y213F^, F22-F2} *[1]. *n *indicates the number of oocyte nuclei observed. *P *values from a *chi *test between the *mei-W68 mus301 *double mutants without any transgene and those with a plant *SPO11 *transgene. ***: *P *< 1E-03; **: 1E-03 <*P *< 1E-02; *: 1E-02 <*P *< 5E-02.

In the negative control experiment, only small fractions of the oocyte nuclei of the *mei-W68*^*1 *^homozygous and *mus301 *hemizygous flies without the transgene showed DSB signals and defective karyosome morphology (7.4% and 1.3%, respectively, Figure 1B and Figure 2A). When the DmSPO11 transgene was expressed under the *Drosophila hsp83 *promoter in the *mei-W68 *(*dmspo11*) *mus301 *double mutant flies, almost all of the oocyte nuclei showed the extensive accumulation of γ-H2Av signals, as in the case of the positive control (the *DmSPO11*-proficient and *mus301-*defective mutant), and the defective karyosome morphology (100% and 70%, respectively, Figure 1C). Thus, we are confident that the γ-H2Av signals in the postmeiotic nuclei of fly oocytes represent the DSBs formed by SPO11.

We then attempted to detect the DSBs induced by a plant SPO11, expressed in *Drosophila mei-W68*^*1 *^homozygous (*dmspo11-*deficient) and *mus301 *hemizygous (*mus301*-defective) double mutant female flies, by an immuno-assay for phosphorylated histone H2Av (γ-H2Av), and by monitoring the number of oocyte nuclei with defective karyosome morphology. The *AtSPO11-1 *or *AtSPO11-2 *transgene was expressed under the control of the *Drosophila hsp83 *promoter in the *mei-W68 *(*dmspo11*) *mus301 *double mutant flies. Both *AtSPO11-1 *and *AtSPO11-2 *are required for meiotic recombination in *Arabidopsis *[21,22]. Thus, we were surprised to find that the expression of either *AtSPO11-1 *or *AtSPO11-2 *significantly increased both kinds of DSB signals in flies, the frequency of γ-H2Av signals and the defective karyosome morphology, as compared with the negative control without the transgene (Figure 1D and 1E; Figure 2A and 2B). These results indicated that both AtSPO11-1 and AtSPO11-2 have the ability to induce DSBs, without the coexpression of other plant proteins, in *Drosophila *oocyte nuclei.

To confirm the expression of transgenes in *Drosophila *oocytes, we performed a quantitative RT-PCR analysis. The expression levels of the transgenes were higher than that of the *Drosophila *ribosomal protein 49 *(RP49) *gene in *Drosophila *oocytes (Table 1).

**Table 1:** Relative levels of mRNA from copies of the plant SPO11 transgene in *Drosophila *ovaries

Insertions of transgenes	Relative levels of mRNA*
*P{hsp83-AtSPO11-1 *cDNA, *A5-1}*	3.40 ± 0.40
*P{hsp83-AtSPO11-2 *cDNA, *2M3-1}*	9.22 ± 2.72
*P{hsp83-OsSPO11A cDNA, 3M2-3}*	13.88 ± 3.59
*P{hsp83-OsSPO11B cDNA, M6-2}*	19.85 ± 4.50
*P{hsp83-OsSPO11B cDNA, F3-2}*	14.57 ± 6.32
*P{hsp83-OsSPO11D cDNA, F2-1}*	3.17 ± 0.36
*P{hsp83-OsSPO11D^Y213F^, F22-F2}*	4.22 ± 0.84
*P{hsp83-OsSPO11D^Y213F^, M33-F1}*	3.76 ± 1.39

* The amount of mRNA from each copy of the plant *SPO11 *transgene in *Drosophila *ovaries was normalized by the amount of mRNA from the *RP49 *gene. The average value with SD for each of the relative levels of transcripts was obtained from three samples, each containing 5~10 ovaries.

### Analyses of disjunction in *Drosophila mei-W68 *(*dmspo11*)-deficient mutants expressing the *SPO11 *transgene

In order to confirm that the expressed plant SPO11 alone introduced DSBs in the chromosomes of *Drosophila *oocyte nuclei, we examined the profiles of X chromosome disjunction caused by *mei-W68 *(*dmspo11*) deficiency in the *Drosophila *DSB-repair proficient (*mus301-*proficient) background, assuming that the unrepaired DSBs caused the X chromosome loss, and the DSBs formed by the plant SPO11 restored the normal X chromosome disjunction in the *dmspo11*-deficient background.

**Figure 3:**
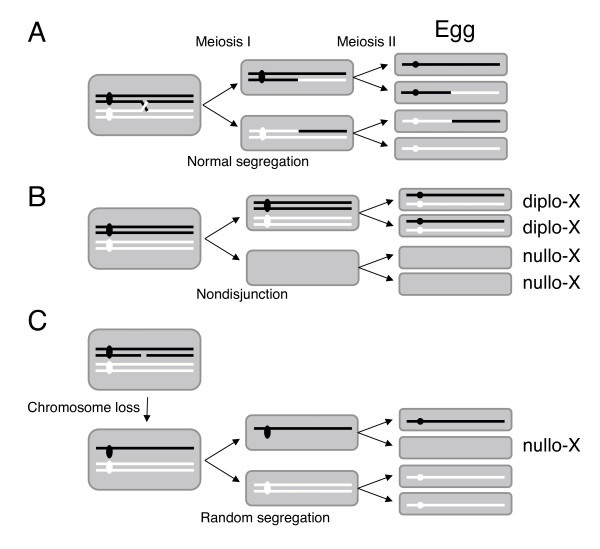
**Aberrant meiotic disjunction of the X-chromosome in *mei-W68 *homozygous (*dmspo11*-deficient) *Drosophila *oocytes induced by plant SPO11s**. **(A) **Normal chromosome segregation in meiosis. Black and white bold lines represent homologous chromosomes, such as X-chromosomes. Each pair of black or white bold lines indicates a parental chromosome duplicated by premeiotic DNA replication, a milestone of the start of meiosis, and each pair remains together until meiosis II. Black and white ovals represent their centromeres. All pairs of duplicated homologous chromosomes (left; indicated as two black and two white lines) are physically connected by at least a chiasma (indicated by a cross) formed by meiotic recombination, and then are segregated into sister cells (middle). After meiosis II, each recombinant or nonrecombinant chromosome is received by each of the four meiotic products (Egg). **(B) **In nondisjunction at meiosis I, one of the sister cells receives both X-chromosomes and the other receives no X-chromosome. In meiosis II, the former sister cell generates two diplo-X eggs, and the latter yields two nullo-X eggs. Thus, each non-disjunction event of the X-chromosome in meiosis I generates two diplo-X eggs and two nullo-X eggs. **(C) **If a DSB(s) is introduced into one of the X-chromosomes, but is not repaired at prophase of meiosis I, then the broken X-chromosome is lost. When random segregation follows in meiosis I, one of the sister cells receives the single X-chromosome and the other receives the duplicated X-chromosome. In meiosis II, the sister cell that received the single X-chromosome generates one nullo-X egg and one normal egg, and the other sister cell that received the duplicated X-chromosome generates two normal eggs. Thus, the X-chromosome aberration in meiosis generates more nullo-X eggs than diplo-X eggs.

The recessive null mutation of *mei-W68 *(*dmspo11*) causes female sterility associated with a high frequency of chromosome nondisjunction in meiosis I, since the crossing-over type of homologous recombination induced by SPO11 is essential for chiasma formation (Figure 3A) [47]. Thus, during meiosis I in mutant cells devoid of meiotic recombination, in half of the cases, one daughter cell receives both paired chromosomes, and the other has none (*i.e*., nondisjunction, Figure 3B). *Drosophila mei-W68*^*1 *^heterozygous (*DmSPO11*-proficient) *mus301-*proficient females displayed 88% fertility, and in contrast, *mei-W68*^*1 *^homozygous (*dmspo11-*deficient) *mus301*-proficient females exhibited 26% fertility (Table 2). The ubiquitous transcription of the *mei-W68^+ ^*cDNA by the *hsp83 *promoter complemented the reduced fertility of the *mei-W68*^*1 *^(*dmspo11*-deficient) homozygotes (from 26% to 87%). However, the expression of the plant SPO11 cDNAs by the same promoter never rescued it (21-24%; Table 2), and thus the DSBs introduced by a plant SPO11 in *Drosophila *are somehow different from those introduced by the host SPO11, Mei-W68 (DmSPO11). As mentioned in the Introduction, the SPO11 protein requires various species-specific interacting proteins for its meiotic function. Thus, it was expected that the expressed plant SPO11s, without their specific interacting proteins in *Drosophila*, would be unable to complement the meiotic defects due to the *mei-W68 *(*dmspo11*) mutation (see Discussion).

**Table 2:** Effects of plant *SPO11 *expression on fertility in the *Drosophila mei-W68*^*1 *^(*dmspo11-*deficient) homozygote

*mei-W68*^*1 *^hetero- zygosity	Transgene	Mothers crossed	Total eggs laid	Pupae generated	% Fertility
+/1	none	37	3768	3309	88
1/1	none	107	6109	1568	26
1/1	*mei-W68*^*+ *§^	11	1207	1047	87
1/1	*AtSPO11-1*^a^	40	2880	691	24
1/1	*AtSPO11-2*^b^	20	1536	359	23
1/1	*OsSPO11D*^c^	60	3577	753	21

Every female (*w/w*) was crossed to a few males of Canton-S strain (*w^+ ^*/Y). The numbers of total eggs laid and the pupae subsequently generated were scored. Fertility was calculated by dividing the number of pupae (representing viable eggs) by the number of total eggs. Transgenes are indicated as *Vector name{gene, insertion #}*.

§: *P{hsp83-mei-W68^+ ^*cDNA, *M53-3}*

a: *P{hsp83-AtSPO11-1 *cDNA, *A5-1}*

b: *P{hsp83-AtSPO11-2 *cDNA, *2M3-1}*

c: *P{hsp83-OsSPO11D *cDNA, *F2-1}*

In meiotic recombination-defective mutants, the nondisjunction of homologous chromosomes in meiosis will theoretically generate an equal number of nullo-X eggs and diplo-X eggs (Figure 3B). A nullo-X egg fertilized with a single X sperm produces a special progeny called an X0 son, and a diplo-X egg fertilized with a single Y sperm produces a progeny called an XXY daughter, when tested females are crossed with normal males with XY chromosomes. The cross of the *mei-W68*^*1 *^homozygous females without the transgene to wild-type males produced progeny that included 11.4% X0 sons, reflecting the proportion of nullo-X eggs to total fertile eggs, and 8.3% XXY daughters, reflecting the proportion of diplo-X eggs to total fertile eggs (Table 3). We interpreted this disparity in the proportion of nullo-X eggs to diplo-X eggs to be the outcome of X chromosome loss caused by spontaneously generated DSBs, occurring with inappropriate timing relative to the progression of meiosis. This interpretation is consistent with the 7.4% background level of DSB signals in *mei-W68*^*1 *^homozygotes with *mus301 *DSB-repair defects. It is further supported by the fact that the X-ray irradiation of cells at meiosis efficiently rescued the crossing-over defect and the homologous chromosome nondisjunction in *mei-W68 *female flies, while the irradiation of the premeiotic and postmeiotic cells did neither [48].

**Table 3:** Effects of plant *SPO11 *expression on X chromosome nondisjunction in the *Drosophila mei-W68^1 ^*homozygote

Transgene of *mei-W68*^*1 *^homozygote	Mothers crossed	Total progeny scored	Nullo-X eggs (%)	Diplo-X eggs (%)	Nullo-X eggs without associated diplo-X eggs (%)	*P *value from *chi *test
Exp. 1						
None	99	1283	146 (11.4)	106 (8.3)	40 (3.1)	-
*AtSPO11-1*^a^	74	1204	138 (11.5)	95 (7.9)	43 (3.6)	5.3E-01
*AtSPO11-2*^b^	71	1187	178 (15.0)	88(7.4)	90 ** (7.6)	6.9E-07
*OsSPO11D*^c^	97	1384	184 (13.3)	106 (7.7)	78 ** (5.6)	1.6E-03

Exp. 2						
none	55	1245	119 (9.6)	88 (7.1)	31 (2.5)	-
*OsSPO11D*^*Y213Fd*^	57	1994	175 (8.8)	129 (6.5)	46 (2.3)	7.4E-01
*mei-W68*^*+ e*^	22	3434	3 (0.09)	3 (0.09)	0 (0)	-

Every female (*w/w*) was crossed to a few males of Canton-S strain (*w^+ ^*/Y). Nullo-X and diplo-X eggs were detected as *w^+ ^*sons (X0) and as *w *daughters (XXY), respectively. The number of nullo-X eggs without associated diplo-X eggs was calculated by subtracting the number of diplo-X eggs from the number of nullo-X eggs. The number of diplo-X eggs was used as the expected value for the number of nullo-X eggs with associated diplo-X eggs, which were generated by chromosome nondisjunction during the first meiotic division. The egg laying periods were 5 days and 10 days in Exp. 1 and Exp. 2, respectively. Transgenes are indicated as *Vector name{gene, insertion #}*.

a: *P{hsp83-AtSPO11-1 *cDNA, *A5-1}*

b: *P{hsp83-AtSPO11-2 *cDNA, *2M3-1}*

c: *P{hsp83-OsSPO11D *cDNA, *F2-1}*

d: *P{hsp83-OsSPO11D^Y213F^, F22-F2}*

e: *P{hsp83-mei-W68^+ ^*cDNA, *M53-3}*

***: *P *< 1E-03; **: 1E-03 <*P *< 1E-02; *: 1E-02 <*P *< 5E-02.

The expression of *AtSPO11-2 *significantly increased X-chromosome loss, as observed by an increase in nullo-X eggs without associated diplo-X eggs (Table 3). The expression of *AtSPO11-1 *increased it slightly (Table 3). These results support the conclusion obtained from the above cytological assays that the expression of either *AtSPO11-1 *or *AtSPO11-2 *actually induces DSBs in the X-chromosomes of oocytes in the absence of functional DmSPO11 in *Drosophila*, but the induced DSBs are not repaired by the normal recombination required for chiasma formation. The DSB forming activity of AtSPO11-1 and AtSPO11-2, as shown by cytological and genetic means, was independent of other plant proteins, such as *Arabidopsis *PRD1, indicating that AtSPO11-1 and AtSPO11-2 express the DSB forming activity either by themselves or with the use of functional *Drosophila *analogues of other plant proteins.

### Identification of a novel SPO11-homologue

Since the bioassay for DSB induction by an expressed *SPO11 *transgene successfully detected the DSB-forming activities of AtSPO11-1 and AtSPO11-2, we then tried to identify the SPO11s that function in DSB formation in the rice *O. sativa*. Candidates of rice SPO11-homologues were cloned from a cDNA library, constructed from RNA isolated from anthers containing meiotic pollens of japonica rice, Nipponbare (see Materials and Methods). OsSPO11A, OsSPO11B and OsSPO11C are almost the same as OsTOP6A, OsTOP6B and OsTOP6C, respectively, of indica rice [20]. The fourth rice SPO11-homologue, AK101363, was found by a homologous sequence search of the database of full-length cDNA clones from japonica rice, KOME (Knowledge-based Oryza Molecular Biological Encyclopedia; http://cdna01.dna.affrc.go.jp/cDNA/), using the amino acid sequence of AtSPO11-1, and consists of 487 amino acid residues.

**Figure 4:**
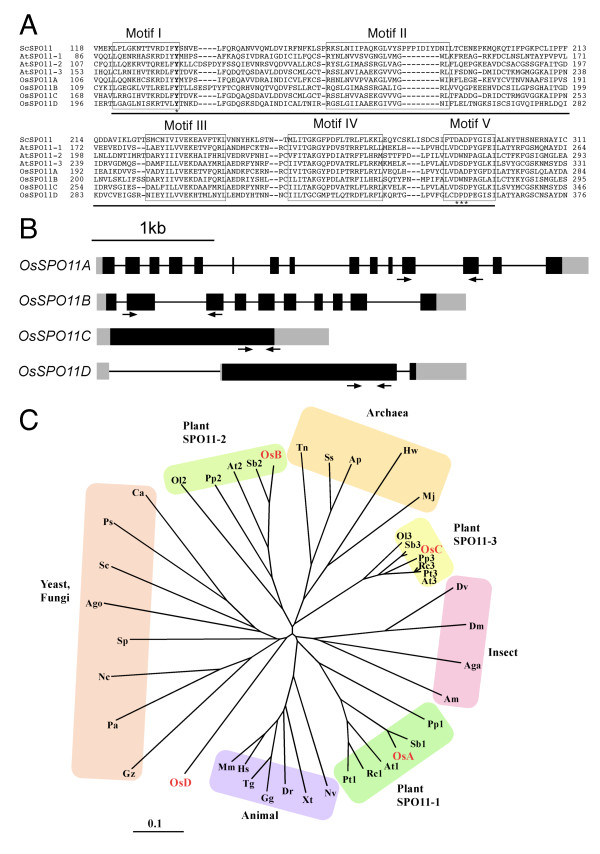
**Comparative analysis of SPO11-homologues**. **(A) **Multiple sequence alignment of motifs I to V of SPO11. The bold tyrosine (Y) residue with an asterisk in motif I is the putative active center for covalent bonding to the 5' termini of cleaved DNA upon meiotic double-stranded cleavage. The triple asterisk in motif V indicates the conserved DXD sequence (see text). Underlined sequences were used for phylogenetic analyses. **(B) **Schematic diagrams of the genomic structures of *OsSPO11 *genes. Black boxes, coding regions; grey boxes, untranslated regions; solid lines, introns. Arrows indicate primers for real-time RT-PCR analyses. **(C) **Phylogenetic analyses of the amino acid sequences of motifs I-V. A neighbor-joining (NJ) tree was constructed using ClustalX ver. 2.0. Aga, *Anopheles gambiae *(EAA05541); Ago, *Ashbya gossypii *(AAS51945); Am, *Apis mellifera *(XP_001122679); Ap, *Aeropyrum pernix *(BAA79679); At1, *Arabidopsis thaliana *(CAB81544); At2, *A. thaliana *(CAB81545); At3, *A. thaliana *(ABI54341); Ca, *Candida albicans *(EAK95423); Dm, *Drosophila melanogaster *(AAC61735); Dr, *Danio rerio *(NP_991245); Dv, *Drosophila virilis *(EDW61133); Gg, *Gallus gallus *(XP_001232076); Gz, *Gibberella zeae *(XP_386125); Hs, *Homo sapiens *(AAD52562); Hw, *Haloquadratum walsbyi *(CAJ52765); Mj, *Methanocaldococcus jannaschii *(Q57815); Mm, *Mus musculus *(AAD52563); Nc, *Neurospora crassa *(CAB88597); Nv, *Nematostella vectensis *(EDO44118); Ol2, *Ostreococcus lucimarinus *(ABO99188); Ol3, *O. lucimarinus *(ABO95960); OsA, OsSPO11A; OsB, OsSPO11B; OsC, OsSPO11C; OsD, OsSPO11D; Pa, *Podospora anserina *(CAP73361); Pp1, *Physcomitrella patens *(EDQ80601); Pp2, *P. patens *(EDQ56207); Pp3, *P. patens *(EDQ71569); Ps, *Pichia stipitis *(XP_001384151); Pt1, *Populus trichocarpa *(EEE85181); Pt3, *P. trichocarpa *(EEE71999); Rc1, *Ricinus communis *(EEF39612); Rc3, *R. communis *(EEF37163); Sb1, *Sorghum bicolor *(EER93438); Sb2, *S. bicolor *(EES14573); Sb3, *S. bicolor *(EER95053); Sc, *Saccharomyces cerevisiae *(AAA65532); Sp, *Schizosaccharomyces pombe *(CAB11511); Ss, *Sulfolobus shibatae *(CAA71605); Tg, *Taeniopygia guttata *(XP_002195778); Tn, *Thermoproteus neutrophilus *(ACB40317); Xt, *Xenopus tropicali*s (AAH80352). The entire sequences of these SPO11 homologues are shown in Additional File 1 Figure S1.

We constructed the full-length cDNAs of all rice SPO11 candidates from the cloned cDNA fragments (see Materials and Methods). Consistent with OsSPO11A, OsSPO11B and OsSPO11C, the amino acid sequence derived from the fourth SPO11-homologue contained all five conserved motifs of SPO11, as well as the conserved tyrosine (tyrosine 135 of *S. cerevisiae*) that is assumed to be essential for the DSB-forming activity in the first motif (Figure 4A) [5,49]. The DxD sequence in motif V, which is proposed to coordinate a Mg^2+ ^cation [49], is also conserved in the fourth SPO11-homologue. Thus, we tentatively named the fourth SPO11-homologue OsSPO11D (DDBJ accession No. AB219540). While OsSPO11A, OsSPO11B and OsSPO11C are very likely to correspond to AtSPO11-1, AtSPO11-2 and AtSPO11-3, respectively, with similarities of 58%, 63% and 69%, OsSPO11D has no *Arabidopsis *counterpart, since it shows low similarity to the *Arabidopsis *SPO11s (20 to 25%), and a BLAST search revealed no similar gene among the genomes of other plants, including *Sorghum *[50] and *Brachypodium *[51], which are both phylogenetically much closer to rice than *Arabidopsis*. Thus, SPO11D is unique to rice.

Figure 4B shows the schematic genomic structures of the four rice SPO11-homologues, which were obtained from the rice genomic sequence in the TIGR rice genome database, using the full-length cDNA sequences as queries. The gene encoding the novel fourth SPO11-homologue, *OsSPO11D*, is 1,572 bp in length and consists of two exons and an intron. This gene structure with few introns is similar to that of *OsSPO11C*, but differs from those of *OsSPO11A *and *OsSPO11B*, which have more than ten exons and introns. We derived the amino acid sequences of the SPO11s of animals, plants, and fungi and the archaeal topoisomerase VI subunit A (TOP6A) from the NCBI web site (Additional File 1, Figure S1). Considering the property that the functionally less important molecules or parts of a molecule evolve faster than the more important ones [52], we focused on Motifs I to V of the SPO11-homologues for phylogenetic analyses by CLUSTALX, and the results are illustrated as a phylogenetic tree (Figure 4C). The previously reported SPO11-homologues of *Arabidopsis *and rice were divided into three phylogenetic groups [53]. OsSPO11D is outside the three groups, and rather close to the fungal SPO11s (Figure 4C). This result suggests the unique origin of OsSPO11D, among the rice SPO11-homologues.

### OsSPO11D is expressed in anthers containing meiotic pollen mother cells

**Figure 5:**
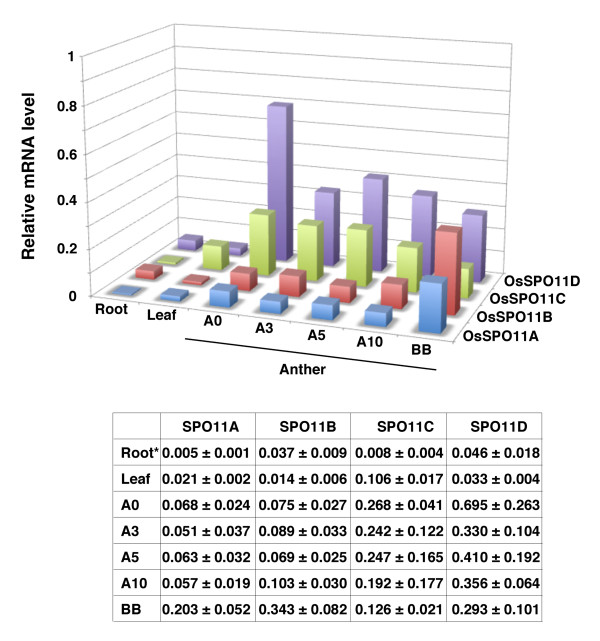
**Quantitative real-time RT-PCR analyses of the amounts of rice SPO11 mRNAs in cells during pollen-development and in organs**. The data shown in the table, representing the accuracy of the data in this figure, were plotted in a 3-dimensional manner for a comparison of their expression profiles. Root, seven day-old root after germination; A0, the anthers in 0 cm DALL (stage A0); A3, the anthers in 3 cm DALL (stage A3); A5, the anthers in 5 cm DALL (stage A5); A10, the anthers in 10 cm DALL (stage A10); BB, before blooming. All data obtained were normalized by the amount of actin gene (AK072796) mRNA, and are expressed as relative mRNA levels, which are the average values with SD obtained from three independent samples.

We then examined the expression of the rice genes encoding SPO11-homologues at the mRNA level, in anthers containing different developmental stages of pollens, from meiotic to mature pollens, as well as in leaves and roots, by the use of quantitative real-time RT-PCR. In rice, the distance between the auricles of the last two leaves (DALL) correlates well with the developmental stage of pollens. The panicle in the 0 cm DALL (stage A0) contains the majority of pollen mother cells at meiosis [54]. The panicle in the 10 cm DALL (stage A10), which is almost two days before heading, contains the mature pollens. The flowers at the BB stage (before blooming) are one day before blooming. The panicles in the 3 cm DALL (stage A3) and 5 cm DALL (stage A5) contain immature to young mature pollens. We isolated the anthers from Nipponbare panicles at the different stages according to the DALL size, and analyzed the expression of the four genes. The results are shown in Figure 5.

The expression patterns of OsSPO11A and OsSPO11B were very similar to each other and rather different from those expected from their meiosis-specific functions; *i.e*., the OsSPO11A and OsSPO11B genes showed lower and constant levels (2-4 fold) of expression in the anthers containing pollens of all stages (A0 to A10), from meiosis to mature pollen, than OsSPO11C and OsSPO11D, although their expression levels were higher than those in the somatic cells, such as leaves and roots. The expression of OsSPO11A and OsSPO11B was induced to a 3-4 fold higher level (10-26 fold of the level in leaves) only at the BB stage, as compared to the other stages of pollen development.

The amounts of *OsSPO11C *mRNA during pollen development from meiosis to the mature pollen stage were within 2.5-fold of that in leaves, and tended to decrease through pollen development, but were generally (except in roots) higher than those of the other *SPO11 *genes, except for *OsSPO11D*. These *OsSPO11C *expression profiles suggest that it might be expressed in the somatic cells of leaves and anthers.

On the other hand, the expression pattern of the *OsSPO11D *mRNA resembled those of genes that are specifically expressed in meiosis; *i.e*., the amount of *OsSPO11D *mRNA increased to a much higher level (*ca*. 20-fold higher than the level in leaves) in the anthers containing meiotic pollens at the 0 cm DALL (stage A0), and subsequently decreased. In the anthers containing meiotic pollens, the amount of Os*SPO11D *mRNA was the highest among the *OsSPO11 *genes; *i.e*., the amount was 4-6-fold higher than those of *OsSPO11A *and *OsSPO11B *(Figure 5). These results support the proposal that, unlike the other *OsSPO11 *homologues, the transcription of *OsSPO11D *is induced specifically and transiently in the meiotic stage of pollen development, suggesting that OsSPO11D plays an important role in rice meiosis.

### OsSPO11D has DSB-forming activity, as detected by the *Drosophila *bioassay

To investigate whether OsSPO11D, OsSPO11A and OsSPO11B can induce DSBs, we measured the DSB forming activity by each of their transgenes, expressed under the control of the *Drosophila hsp83 *promoter, in the *mei-W68 *(*dmspo11*) *mus301 *(DSB-repair defective) double mutant flies. The expression of OsSPO11D in the flies generated large numbers of oocyte nuclei with both kinds of DSB signals, the γ-H2Av signals and the defective karyosome morphology (Figures 1G and 2). In order to confirm that OsSPO11D directly introduced DSBs in *Drosophila *oocytes, we expressed a mutant transgene, *OsSPO11D^Y213F^*, with the phenylalanine-substitution of the 213th tyrosine in the putative catalytic center, under the control of the *Drosophila hsp83 *promoter. The *OsSPO11D^Y213F ^*transgenes were expressed at a similar level as the wild-type *OsSPO11D *transgene (Table 1), but never showed any additional DSB activity over the background level, in terms of DSB signals (Figure 2A) and karyosome morphology (Figure 2B). Thus, we conclude that OsSPO11D has intrinsic DSB-forming activity.

The expression of OsSPO11A in flies generated almost the same levels of DSB signals and defective karyosomes as AtSPO11-2 (Figures 1E, 1F and 2). On the other hand, the expression of OsSPO11B in flies did not generate any significant differences in the DSB signals and karyosome morphology over the negative control (without transgene), unlike the case of its *Arabidopsis *counterpart, AtSPO11-2 (Figure 2). In addition, the co-expression of OsSPO11A and OsSPO11B in flies showed no additive or synergistic effect on the generation of DSB-signals in fly oocytes (Figure 2). These results indicate that OsSPO11A and OsSPO11D have the ability to induce DSBs without the coexpression of other plant proteins in *Drosophila *oocytes.

To confirm the DSB activity of OsSPO11D in *Drosophila*, the nondisjunction test was performed. The expression of OsSPO11D in the *mei-W68*^*1 *^homozygous (*dmspo11*-deficient) *mus301*-proficient flies increased the proportion of nullo-X eggs without associated diplo-X eggs by 5.6% from the control level (3.1%; Table 3; P < 1E-02 by *chi *test). The catalytic mutant transgene of OsSPO11D showed no increase in the proportion of nullo-X eggs without associated diplo-X eggs (Table 3). From these results, we conclude that OsSPO11D, like AtSPO11-1 and AtSPO11-2, has DSB forming activity.

Finally, to test whether the OsSPO11D-induced DSBs are repairable, we examined the DSB signals in *mei-W68*-deficient, but *mus301*-proficient, flies with the *OsSPO11D *transgene under *hsp83 *promoter control (*P{hsp83-OsSPO11D cDNA, F2-1}*). Among 116 oocyte nuclei (stages 2-8), only 2 nuclei showed γ-H2Av signals, and no defective karyosomes (stages 3-8) were observed (0/94), suggesting that the OsSPO11D-induced DSBs are joined by a DSB-repair mechanism that does not allow chiasma formation.

## Discussion

Plants have multiple functional SPO11 candidates (see Introduction), and the SPO11 candidate(s) responsible for meiotic DSB formation has not been identified. To answer this question, we developed a *Drosophila *bioassay for the quantitative evaluation of the DSB activity of SPO11 candidates expressed from transgenes in fly oocytes. The interspecies bioassay that we developed in this study was effective to detect the DSB signals generated by the trans-expression of SPO11s and to identify the SPO11s with DSB-forming activities, among the candidates in plants. It was shown for the first time that both AtSPO11-1 and AtSPO11-2 exhibit DSB activity alone, in the absence of species-specific (*i.e. Arabidopsis*) interacting proteins for SPO11 functions. *Arabidopsis *has three genes encoding SPO11 homologues. Two of them (AtSPO11-1 and AtSPO11-2) are required for meiosis. Our results are surprising, since previous genetic studies showed that both AtSPO11-1 and AtSPO11-2 are required for meiosis, and interacting proteins, such as PRD1, are required for the SPO11 functions (see Introduction section). Note that our results do not mean that AtSPO11-1 and AtSPO11-2 work independently in *Arabidopsis*. It is likely that in *Arabidopsis*, SPO11-interacting proteins coordinate AtSPO11-1 and AtSPO11-2 to induce functional meiotic DSBs, leading to chiasma formation.

By using this method, we further investigated the three rice SPO11 homologues and a novel one, OsSPO11D. OsSPO11A and OsSPO11B are considered as the counterparts of *Arabidopsis *AtSPO11-1 and AtSPO11-2, respectively, from their amino acid-sequence similarities. The bioassay revealed that OsSPO11A has DSB activity, as in the case of its *Arabidopsis *counterpart (Figure 2). However, we obtained another unexpected result from other tests: our assay did not detect any significant DSB-forming activity of OsSPO11B, unlike the case of its *Arabidopsis *counterpart, AtSPO11-2 (Figure 2). This was surprising, since OsSPO11B was expressed in the fly at a similar level as OsSPO11A, and at two- to five-fold higher levels than AtSPO11-1, AtSPO11-2 and OsSpo11D, which all showed DSB signals in the oocyte nuclei (Table 1 and Figure 2). This observation does not support the suggestion from the phylogenetic analyses (Figure 4) that OsSPO11B is an orthologue of AtSPO11-2. OsSPO11D displayed robust DSB activity (Figures 1G and 2).

All of the results obtained from the analysis of the γ-H2Av signals were supported in parallel by the analysis of the frequencies of oocyte nuclei with defective karyosome morphology in the DSB-repair defective fly, which represent the ectopically expressed plant SPO11-induced DSBs in *Drosophila *oocyte nuclei (Figure 2).

In order to obtain additional evidence for the DSB activity of SPO11s, we performed genetic tests, using the *dmspo11*-deficient, but *mus301*-proficient (DSB-repair proficient), flies. We assumed that if the DSBs induced by a plant SPO11 are not repaired, then the DSBs caused the X chromosome loss in *Drosophila *oocytes, and if they are repaired by a normal recombination process leading to chiasma formation, then the DSBs restored the normal meiotic X chromosome disjunction. The expression of DmSPO11 from the transgene fully restored the meiotic deficiencies of the *dmspo11 *mutant flies (Table 2). The expression of the rice SPO11 transgenes, as well as the *Arabidopsis *SPO11s, in oocytes bearing the *dmspo11 *mutation caused an increase in nullo-X eggs, without associated diplo-X eggs (Table 3). The aberrant X-chromosome segregation, which was induced by the expression of the OsSPO11D, AtSPO11-1 and AtSPO11-2 transgenes in *dmspo11 *mutant flies, correlated well with the abilities of the expressed OsSPO11D, AtSPO11-1 and AtSPO11-2 proteins to induce DSBs, as shown by the γ-H2Av signals in the oocyte nuclei of the *dmspo11 mus301 *double mutant flies (Table 3 and Figure 2). This profile is explained by the X chromosome abnormalities caused by aberrant DSBs, as shown in Figure 3C. Therefore, we conclude that OsSPO11D, as well as AtSPO11-1 and AtSPO11-2, has DSB activity in oocytes.

Then, one may wonder why the DSBs induced by the plant SPO11s did not complement the meiotic defect of the *dmspo11 *mutant flies. The importance of the timing of DSB formation in meiotic disjunction was clearly shown by Bhagat *et al*. [48]: X-ray irradiation of prophase I oocytes of *dmspo11*-deficient mutant flies efficiently induced meiotic exchanges and suppressed meiotic nondisjunction of the mutant flies. However, pre-meiotic or post-meiotic irradiation did not induce meiotic exchange and caused more severe nondisjunction in the mutants. The *S. cerevisiae *Spo11 protein, expressed by the ubiquitous *ADH1 *promoter in the host, exhibited DSB activity only in meiotic cells [55]. In addition, the DSB activity of Spo11 required meiosis-specific and meiosis-nonspecific interactors [56]. These findings indicated the presence of regulatory factors for Spo11 to exhibit the proper timing of the DSB activity. In this study, we found that *DmSPO11*, expressed by the ubiquitous *hsp83 *promoter in the *Drosophila dmspo11 *mutant, allowed normal progression through meiosis. This suggested that, in *Drosophila*, a regulator conferring the stage specificity of DmSPO11-induced DSB formation prevents the DNA scission by the DmSPO11 protein in the premeiotic or postmeiotic stage, but in early pachynema, another regulatory factor induces the active form of the DmSPO11 complex, leading to DSB formation for crossing-over. It is likely that the *Drosophila *regulatory factors are unable to regulate the *Arabidopsis *SPO11s. Thus, the DSB induction by plant SPO11s, expressed by the ubiquitous promoter *hsp83 *in the absence of plant SPO11-interacting protein factors, is unregulated and causes untimely DSBs in *Drosophila *oocytes. The DSBs induced at inappropriate times are repaired in the *mus301*-proficient fly as described, probably through inter-sister chromatid-homologous recombination or non-homologous end-joining, rather than inter-homologous chromosome-recombination, which is required for chiasma formation.

A recent paper reported that RNA interference for OsSPO11D (described as OsSPO11-2 in this paper) reduced pollen viability and seed setting rates in rice [57]. Our finding of the specific expression of *OsSPO11D *at the transcriptional level in the anthers containing meiotic pollens (Figure 5) further strengthens the specific role of OsSPO11D in meiotic recombination, as in the case of SPO11 in the yeast *S. cerevisiae*, which is expressed specifically at meiosis I under strict transcriptional regulation [58].

More studies on OsSPO11D are necessary to prove this hypothesis. The bioassay described in this study would also be helpful to identify species-specific SPO11-interacting proteins that stimulate the SPO11 functions, the DSB forming activity specific for prophase I pachytene chromosomes, and the following interactions with repair enzymes. The biochemical characterization of purified SPO11s is necessary. We purified AtSPO11-1 [32], and other plant SPO11s (Y. S. unpublished observations) in soluble forms, but they lacked detectable endonuclease activity. A recent report claimed that OsSPO11D exhibited DSB forming activity by itself *in vitro *[57]. Further studies are needed to determine whether the purified SPO11s introduce the DSBs with either site- or sequence-specificity and are attached covalently at the termini of the cleavage sites, and which amino acid substitutions inactivate the SPO11s.

## Conclusions

This study showed that, in the absence of other plant proteins, the *Arabidopsis *SPO11s required for meiosis, AtSPO11-1 and AtSPO11-2, and the rice AtSPO11-1 counterpart, OsSPO11A, exert the DSB activities by themselves or with the use of *Drosophila *functional analogues of other plant proteins. The *Drosophila *bioassay revealed a novel rice SPO11 homologue, OsSPO11D, which was suggested to be a functional SPO11 in rice meiosis, along with OsSPO11A.

## Methods

### Cloning of rice SPO11 cDNAs and plasmid constructions

Based on the DNA sequences of the Arabidopsis *AtSPO11-1, AtSPO11-2 *and *AtSPO11-3 *genes, four *OsSPO11 *gene candidates were identified from the rice cDNA database http://cdna01.dna.affrc.go.jp/cDNA/. The *rice SPO11s *corresponding to *AtSPO11-1, AtSPO11-2 *and *AtSPO11-3 *were named *OsSPO11A, OsSPO11B *and *OsSPO11C*, respectively. The fourth *OsSPO11 *was designated as *OsSPO11D*. All *OsSPO11s *were amplified by RT-PCR, using RNA isolated from anthers containing meiotic pollens and the primers listed in Additional File 2, table S1. The *OsSPO11C *cDNA was cloned as a single DNA, and the other *SPO11-*homologue genes were amplified as two overlapping 5' and 3' fragments (Additional File 2, table S1). The products were inserted into the pCR2.1 plasmid, using a TA Cloning^® ^Kit (Invitrogen, Carlsbad, CA), and were sequenced. To obtain the whole genes, the *Eco*RI, *Aat*II and *Cla*I restriction sites were used for *OsSPO11A, B *and *D*, respectively, with the appropriate restriction sites on pCR2.1 (Additional File 2, table S1). To create the *Drosophila *transformants, we generated a P-vector bearing the *Drosophila hsp83 *promoter, for the expression of the cloned gene in germ-line cells. The 0.9-kb *hsp83 *promoter genomic region was amplified by PCR, using genomic DNA from the wild-type strain Canton-S and the following modified primers to create the *Xho*I (5') and *Hpa*I (3') restriction sites: 5'-GGGCTCGAGG GGAACTTGAA GAAGTGCATA TTGGGG-3' and 5'-CCCGTTAACC AGACGCTGCT TGTTGTTACG ACGC-3', respectively [59]. The 0.9-kb *Xho*I/*Hpa*I fragment was then purified. The P-vector pCasper-hs, which is generally used for heat-shock induced expression of transgenes, was digested with the *Xho*I and *Hpa*I restriction enzymes to remove the *hsp70 *promoter region, and the 8.4-kb *Xho*I/*Hpa*I fragment was then ligated with the 0.9-kb *Xho*I/*Hpa*I fragment to form the new vector, pCasper-hsp83. The cDNAs encoding *AtSPO11-1, AtSPO11-2, OsSPO11A, OsSPO11C*, and *OsSPO11D *were re-amplified with the primers listed in Additional File 2, Table S1 and digested with *Bgl*II or *Bam*HI and *Kpn*I. These fragments were inserted into pCasper-hsp83 at the *Bgl*II-*Kpn*I sites. The mutant *OsSPO11D *cDNA, harboring the single amino acid substitution (Tyr 213 to Phe), was constructed by using a QuikChange Site-Directed Mutagenesis kit (Stratagene, La Jolla, CA, USA), and the primer pairs are listed in Additional File 2, table S1.

### Genetic techniques

*Drosophila *stock construction and maintenance were performed on standard cornmeal-molasses medium at room temperature (22-25°C). All *Drosophila *crosses for tests were performed at 25°C. Mutations and abbreviations are described in FlyBase [60]. The chromosomes used are as follows: the mutagen-sensitive mutant *mus301^D4^*, the deficiency uncovering the *mus301 *locus *Df(3L)66C-G28*, and the meiotic mutant *mei-W68*^*1 *^(Bloomington Stock Center). The balancer chromosomes were *CyO *and *TM3 *(Kyoto Stock Center).

### *Drosophila *cytological techniques for ovary fixation and immunofluorescence

Virgin females were aged for 4 to 5 days at 25°C, and then their ovaries were dissected in 1 × PBS. The ovaries were transferred to a microcentrifuge tube containing 4% paraformaldehyde in 1 × PBS and fixed for 30 min to 1 hr at room temperature. The fixative was carefully removed, and the ovaries were washed in 0.5 ml of CSC buffer (10 mM citric acid/sodium citrate buffer (pH 6.0)) supplemented with 0.1% (v/v) Igepal CA-630 (Sigma no. 18896), which was used as a nonionic detergent in place of Triton X-100. The ovaries were then denatured for 20 min at 85°C, in 1.2 ml of CSC buffer supplemented with 0.1% (v/v) Igepal CA-630 [61]. The tissue was rinsed in 1 ml of PBSI-BSA (1 × PBS + 0.1% Igepal CA-630 + 5% BSA) and incubated at room temperature for 30 min with 10 μg/μl RNaseA in PBSI-BSA. The tissue was rinsed in PBSI-BSA, and then blocked for 1.5 hr at room temperature in PBSI-BSA. Antibody staining was performed as described previously [62]. The first antibody, a mouse monoclonal anti-human phospho-H2AX (Ser139) antibody that cross-reacts with *Drosophila *γ-H2Av (1:100 dilution; Upstate Biotechnologies, Lake Placid, NY), was added in PBSI-BSA and incubated for 48 hrs at 5°C. The tissue was washed in PBSI (1 × PBS + 0.1% Igepal CA-630). The secondary antibody, donkey anti-mouse IgG conjugated with Alexa-555 (Molecular Probes, Eugene, OR), was diluted at 1:100 in PBSI-BSA and incubated for 16 - 24 hrs at 5°C. DNA staining was performed in the solution for the secondary antibody reaction, using OliGreen™ (Molecular Probes, Eugene, OR). Sample images were collected on a confocal microscope with a OLYMPUS FLUOVIEW FV1000, using the OLYMPUS FV10-ASW[Ver1.6] program, and adapted using the Photoshop 5.0 LE software (Adobe).

### *Drosophila *female fertility and X-chromosome nondisjunction assays

For the determination of the female fertility and the X-chromosome nondisjunction frequencies, each of the tested females carrying the *white *mutant allele on the X-chromosome was crossed to a few wild-type males with the *white^+ ^*allele. After 2 days of egg laying, each cross was transferred to a new food vial every day. The number of eggs laid was scored, and subsequently, the number of pupae generated was scored. Fertility was determined by calculating (pupae)/(total eggs). X-chromosome nondisjunction produces nullo-X eggs and diplo-X eggs. Nullo-X eggs were detected as *white^+ ^*sons (X0 males) and scored. Diplo-X eggs were indicated by *white *mutant daughters (XXY females) and scored. The frequencies of nullo-X eggs and diplo-X eggs were calculated as the proportions of the numbers of X0 males and XXY females to total progeny, respectively.

### Quantitative real-time PCR of transgenes in *Drosophila *oocytes

For the expression analysis of transgenes, ovary tissue was isolated from *Drosophila*. Total RNA was isolated by using Isogen (Nippon Gene, Tokyo, Japan). Quantitative real-time PCR was performed with CHROMO4 (Bio-Rad Laboratories, Hercules, CA, USA), using the PCR primers listed in Additional File 2, table S1.

### Phylogenetic tree

Multiple alignments of the deduced amino acid-sequences of SPO11-homologues were accomplished by using the ClustalX program [63]. The alignments were created with the program ClustalX 2.012, using the Gonnet series matrix (Gap Opening, 10; Gap Extension, 0.2; Delay Divergent Sequences, 30%; Negative Matrix, off), and then were automatically converted to tree files with the Neighbor-Joining method. The results were visualized using the TREEVIEW software http://taxonomy.zoology.gla.ac.uk/rod/treeview.html.

### RNA extraction and quantitative real-time RT-PCR of SPO11-homologues

For the expression analysis of SPO11-homologues, panicles and leaves were harvested from rice plants (*Oryza sativa *L. var. Nipponbare) grown in pots under natural light conditions during the summer. Roots were harvested from Nipponbare seedlings grown on Murashige and Skoog's medium [64] for 7 days. The anthers were collected from each panicle, and the leaves and roots were frozen immediately in liquid nitrogen and then stored in a freezer (-80°C) until use. Total RNA was isolated from anther, leaf and root tissues by using a TRIzol Plus RNA Purification kit (Invitrogen), according to the manufacturer's instructions. Reverse transcription was performed by using the Superscript^® ^III first strand system (Invitrogen). For quantitative real-time RT-PCR experiments, PCR primers (19-24 bp, Additional File 2, table S1) were designed to amplify 70-118 bp fragments with a melting point range from 75-85°C, following Perkin-Elmer's recommendations for SYBR Green primers. Quantitative real-time RT-PCR was performed with an ABI Prism 7000 Sequence Detection System (Perkin-Elmer Applied Biosystems, Foster City, CA), using Power SYBR^® ^Green PCR Master Mix (Applied Biosystems), in 25 μl reaction mixtures containing the cDNA template and 50 nmol primer. The standard two-step thermal cycling protocol was repeated 40 times, and then the melting points of the amplified products were determined with the prescribed dissociation protocol, to verify that the signal was derived from a single amplicon.

## List of abbreviations

DSB: double-stranded break; RT-PCR: reverse transcriptase polymerase chain reaction; DALL: the distance between the auricles of the last two leaves; SD: standard deviation.

## Authors' contributions

YS performed the molecular genetic studies, including the construction of the full-length cDNAs and their expression vectors, assisted with the sequence alignment and helped to write the manuscript. TT performed the molecular genetic studies, especially the RT-PCR analysis of the rice *SPO11 *mRNAs, and assisted with the sequence alignment. YA performed cytological and genetic studies on *Drosophila*. KT and SA performed the molecular genetic studies on rice. MK and AK participated in the isolation of mRNA from rice meiotic tissues and their cDNA libraries. TM assisted with the design of the expression vectors of plant *SPO11 *cDNA and helped to write the manuscript. MY participated in the design of the *Drosophila *bioassay. KW assisted with the design of rice genetic experiments, performed their genetic analyses, and helped to write the manuscript, especially the part about the rice molecular genetic studies. TS participated in the design of the study and its coordination, and helped to write the manuscript. KK designed and performed the *Drosophila *bioassay, and participated in coordination and writing the manuscript. All authors read and approved of the final manuscript.

## Supplementary Material

Additional file 1 **Supplementary Figure S1**. Multiple alignments of conserved sequences of the SPO11 proteins for phylogenetic analyses. These alignments were constructed using the CLUSTALW program at DDBJ. Asterisks indicate invariant amino acids. For the species abbreviations, see the legend of Figure 4C. Click here for file

Additional file 2 **Supplementary Table S1: Primer sequences (5' to 3')**. Click here for file
