# Supplementary material for: The double-stranded break-forming activity of plant SPO11s and a novel rice SPO11 revealed by a Drosophila bioassay
Source: BMC Mol Biol. 2012 Jan 16;13:1. doi: 10.1186/1471-2199-13-1 (PMC3273433; doi:10.1186/1471-2199-13-1)
Supplement: Additional file 1 — Supplementary Figure S1. Multiple alignments of conserved sequences of the SPO11 proteins for phylogenetic analyses. These alignments were constructed using the CLUSTALW program at DDBJ. Asterisks indicate invariant amino acids. For the species abbreviations, see the legend of Figure 4C. [file 1471-2199-13-1-S1.PDF]

```
Mj(89)    LLETDDFSTLR-----EAYYVSKNWG--------------EARFDDQQASNNVIEDLEAALGVL--REHLGFIPEEDG--SSVVGPLKIIEETPEGEL----VVDCTK---LGTGAYNIP(178)
At3(157)  LCLRNIHVTKR-----DLFYTDVKLFQ-----------------DQTQSDAVLDDVSCMLGCT--RSSLNVIAAEKG---VVVGRLIFSDNG----D----MIDCTK---MGMGGKAIP(237)
Pt3(152)  LCLKSIHVTKR-----DLFYTDVKLFQ-----------------DQTQSDAVLDDVSCMLGCT--RSSLNVIAAEKG---VVVGRLIFSDNG----D----MIDCTK---MGMGGKAIP(232)
Rc3(150)  LCLKNIHVTKR-----DLFYTDVKLFQ-----------------DQTQSDAVLDDVSCMLGCT--RSSLNVVAAEKG---VVVGRLIFSDNG----D----MIDCTK---MGMGGKAIP(230)
Pp3(142)  LCTKQIHVTKR-----DLFYTDVKLFQ-----------------EQGQSDTILDDVSCMLGCT--RSSLNVVASEKG---VVVGRLIFIEDG----D----RIDCTK---MGVGGKAIP(222)
OsC(172)  VLRRGIHVTKR-----DLFYTDVKLFG-----------------DQAQSDAVLDDVSCMLGCT--RSSLHVVASEKG---VVVGRLTFADDG----D----RIDCTR---MGVGGKAIP(251)
Sb3(169)  VLGRGIHVTKR-----DLFYTDVKLFG-----------------DQSQSDAVLDDVSCMLGCT--RSSLHVVASEKG---VVVGRLVFADDG----D----RIDCTR---MGVGGKAIP(249)
Ol3(68)   VLSRGIHVTKR-----DLFYTDVKLFR-----------------DQKDSDAVLDDVSCMLGCT--RNSLHVVASEKG---VVVGRLQYKEDG----D----EIDCTK---MGVGGKAIP(148)
OsB(112)  ILGEGKLVTLR-----ELFYTLLSESP--------------TYFTCQRHVNQTVQDVFSLLRCT--RQSLGIMASSRG---ALIGRLVVQGPEE---E----HVDCSI---LGPSGHAIT(197)
Sb2(111)  ILGEGKLVHQR-----ELFYKLLSDSP--------------KYFSCQRHVNQAIQDVVSLLRCT--RQSLGVMASSRG---ALIGRLVLHEPDE---E----QIDCSI---LGASGHAIT(196)
At2(110)  ILLQEKRVTQR-----ELFYKLLCDSP--------------DYFSSQIEVNRSVQDVVALLRCS--RYSLGIMASSRG---LVAGRLFLQEPGK---E----AVDCSA---CGSSGFAIT(195)
Pp2(11)   LLGIGKQATQR-----EIFYNILSTSS--------------SYITSQNQVNSAIQDAVAVLLCT--RRSLGILASSKG---AVVGRLVIKEHGG---D----TVDCSN---LGSSSYPIS(96)
Aga(12)   LLTTGSSCTKR-----ELYY-LHLELAQ----------------TPAYTYATLEDVCALLDADP--WELHVYNTSKG---LIAGPIVLTLSDG---Q----RIDCNG-----RWGTAVP(91)
Am(132)   LLITNTTLTRR-----SLYYDLKNENTSNLVP------------EQRCLDQAVHHVANLLNCAP--WDLNLLPTSKG---LVAGELTLTLADN---R----VIDCTVP------GGALIP(216)
Ol2(2)    ---------LR-----ELYYVMNSRGRRGGWPSGSTQTRV----TEHQIAATIRKISQALGAP--RIALGIVAASKG---QVAGRCHIETASG---A----RFDCTA---CGNDGWAIT(88)
Dm(67)    LQVRGGSFTVR-----GLYYDNPLLVR-----------------SQSRIAEARLDVCRMLRTS--PLSLGILAASKG---LVAGDLRLLMTNG---D----VLDSSL----YGGPLTLP(147)
Dv(85)    LHLSGGSCTIR-----GLYYRDTHVVR-----------------SQSYIVAAKLDVCRMLNTA--PVNLGILSASKG---LIAGDIKLLMSNG---D----ILDCNV----YCGAITLP(165)
Ap(93)    SLIENEYPTIR-----DLYYKGKHTIVYRDYSGR---KREENTWDEQKESDSVIQDIEVYTGLF--REDMLILSKEK---GKVVGNMRIRSGG---D----VIDLSK---LGHGAYAIE(188)
Ss(92)    ALVSDEYPTIR-----DLYYRGKHSLLLKSIEGNKI-VSEENTWDEQKESDSVIVDIEVFTSLL--REEMLILSKEK---GKVVGNLRIRSGN---D----VIDLSK---TGHGAYAIE(189)
Tn(82)    AIREDVYPTIR-----DLYYNGKHTITFKDPLGR---THRENTWDEQAESNAVIEDIEVAANVL--REEMGVSADVK---GKIVGPIVVRSQG---Y----ELDASK---FGETALSLP(177)
Hw(85)    QLEEDRSSTLR-----ELYYLSESWDNK-----------EAQFSDQDESNQLVEDLEIVSKVT--REDFHMRPEESG--ATLMGPLELREQTRRGER----AIHCQED--VGEGGYQIP(177)
Dr(114)   LVQSDSYATKR-----DIYYNDPQLFG-----------------SQRMLDSIVDDISCMLKVP--RRSLHILATSKG---FISGDLCYLEEDG---T----KVDCNS----SSTAVSVS(191)
Xt(124)   LVQTDTYTTKR-----DIYYNDVQLYG-----------------SQTVVDNIVSDLSCMLKIP--RINLHILSTSKG---CVAGDLWFTAEDG---S----KVYCGG----SSSGVLVP(204)
Mm(124)   LIQSDTYATKR-----DIYYTDSQLFG-----------------NQAAVDSAIDDISCMLKVP--RRSLHVLSTSKG---LIAGNLRYMEEDG---T----RVQCTC----SATATAVP(204)
Nv(133)   LLQEDRFATKR-----DVYYTDTVFFG-----------------NQNVVDDIVDNLSCMLEVP--RHSLRILASSKG---LISGHVRYREYDG---T----YVDCTC----LTRGVAVS(213)
Nc(137)   ALLSGTLVTKR-----NIYYQNMELFR-----------------SQSVVDDMVDNLAFTLGVG--RNDLNIVATAKG---LVAGQVELIMRGG---S----KIDCAES----SDSATFR(217)
Pa(118)   ALLSGMLITKRSVLIRNIYYQSPDLFG-----------------SQAAVDVMVDNLAFTLGVG--RGDLNIVATAKG---LISGPFMLISRDG---S----VIDCGLS-----HSTGIL(202)
OsD(199)  TLGAGLNISKR-----TVLYTNKDLFG-----------------DQSKSDQAINDICALTNIR--RGSLGIIAAEKG---IVVGNIFLELTNG---K----SISCSIG-----VQIPHR(278)
Sp(84)    AVISDTVITKR-----DIYYRDVDLFKR-----------------QTVVDELLGDISNTIGCS--RSDLNVEASAKG---LVFGSIHIALENG---T----VITATKP---------LL(159)
Ago(109)  RMRSRQTATVR-----DVFYGNVELYG-----------------RQQVVVDWLVALEQCFGVEK--SVFRILAAQKG---LVHVPVPLEVEG-----------QELKG----VGLIPYV(185)
Sc(121)  KLPLGKNTTVR-----DIFYSNVELFQR-----------------QANVVQWLDVIRFNFKLSP--RKSLNIIPAQKG---LVYSPFPIDIYDNILTCENEPKMQKQTIFPGKPCLIPFF(213)
Gz(115)   ALVSGTVLTKR-----HIFYQHQDLFE-----------------KQREVDDLVDDIAFTLGIS--RGDLNIVAASKG---VLAGPLTIGLHDG---ST----LNPCLG-----DLILIG(194)
Ca(51)    LKAQNQITTIR-----DIYYQDVEVFN-----------------HCQNECRFLLGQLVERGLGWSLCDDLNIHPTQKG---LVYGDYFQ--------------------ELSIKAEPILIPIN(128)
Ps(129)   QSNSKKSTTIR-----DIYYQDVEAFH-----------------WKQRYCNEILHSIVVDSLGLSLEHNFSIYPSQKG---LVYGDFAIQSNEGTI-F--------QMSYSEEPVLIPL-(213)
Pp1(11)   LLQQNRHATKR-----DVYYNDTALLKG------Q--------MVIYSRLDQSINDICCFFHCS--RPSLNLATYAMRGSSLVTGQLSYYEEG---R----LINCSKR-SSTSNGLPVP(100)
Gg(136)   MVQSNSYATKR-----DIYYSDKLLFG-----------------SQRVVDNLINEISCMLQIP--RRSLHILSTTRG---FVAGNLSYTEEDG---T----KVNCTC----GATAVTVP(216)
Sb1(111)  LLQQNKHCSKR-----DIYYMYPSIFV-----------------EVAVVDRAINDICILLKCS--RHNLNVVPVVKG---LVMGWIRFVEGE----K----KVYCIT---NVNAAFSIP(191)
Hs(124)   LVQSNTYATKR-----DIYYTDSQLFG-----------------NQTVVDNIINDISCMLKVS--RRSLHILSTSKG---LIAGNLRYIEEDG---T----KVNCTC----GATAVAVP(204)
Tg(89)    MVQSNTYATKR-----DIYYSDTLLFG-----------------SQSVVDQIINDISCMLKIP--RGSLHILSTTKG---FVAGNLSYTEEDG---T----KVNCTC----SATAVTVP(169)
Pt1(86)   LLQENRHGSKR-----DIYYMHPSVFS-----------------EQSVVDRAINDICILLQCS--RHNLNVVSVGNG---LVMGWLQFLEAG----R----KFDCIS---SPTTAYTIP(166)
OsA(109)  LLQQNKHCSKR-----DIYYMYPSIFQ-----------------EQAVVDRAINDICVLFKCS--RHNLNVVPVAKG---LVMGWIRFLEGE----K----EVYCVT---NVNAAFSIP(189)
Rc1(89)   LLQENKHGSKR-----DIYYMHPSVFS-----------------DQSVVDQAINDICIILQCS--RHNLNVVSVGTG---LVMGWLRFLEAG----R----KFDCIN---SPNNVHPIP(169)
At1(89)   LLQENRHASKR-----DIYYMHPSAFK-----------------AQSIVDRAIGDICILFQCS--RYNLNVVSVGNG---LVMGWLKFREAG----R----KFDCLN---SLNTAYPVP(169)
                   *

Mj    N-DVTKLNLET----DADFILAIETSGMFARLNAERFWDKHN-----------CILVSLKGVPARATRRFIKRLHEEH-------DLPVLVFTDGDPYGYLN(257)
At3   P-NIDRVGDMQS---DAMFILLVEKDAAYMRLAEDRFYNRFP-----------CIIVTAKGQPDVATRLFLRKMKMEL-------KLPVLALVDSDPYGLKI(317)
Pt3   P-NIDRVGDMQS---DALFILLVEKDAAYMRLAEDRFYNRFP-----------CIIVTAKGQPDVATRLFLRKMKTEL-------KLPVLALVDSDPYGLKI(312)
Rc3   P-NIDRVDDMQS---DALFILLVEKDAAYMRLAEDRFYNRFP-----------CIIVTAKGLKGQPDVATRLFLRKMKMEL----KLPVLALVDSDPYGLKI(310)
Pp3   P-NIDKVQGMES---DALFILLVEKDAAFMRLAEDRFYNRFP-----------CVILTAKGQPDVATRLFLKKMKTDL-------KLPVLGLVDSDPYGLKI(302)
OsC   P-NIDRVSGIES---DALFILLVEKDAAFMRLAEDRFYNRFP-----------CIILTAKGQPDVATRLFLRRLKVEL-------KLPVLALVDSDPYGLKI(331)
Sb3   P-NVDRVSGIES---DALFILLVEKDAAFMRLAEDRFYNRFP-----------CIILTAKGQPDVATRLFLRRLKVEL-------KLPVLALVDSDPYGLKI(329)
Ol3   S-NIDKITDMRT---DAKFVLLVEKDAAFMRLAEDRFYNKYP-----------CIIITAKGQPDVATRLFLSKVKRDL-------KIPVLALVDADPYGLKI(228)
OsB   GDLNVLSKLIFSS--DARYIIVVEKDAIFQRLAEDRIYSHLP-----------CILITAKGYPDLATRFILHRLSQTY------PNMPIFALVDWNPAGLAI(280)
Sb2   GDLNLLSKLNLSS--DARYIIVVEKDAIFQRLAEDRLYNQLP-----------CILITAKGYPDIATRFILHRLSQTF------PNMPIFALVDWNPAGLAI(279)
At2   GDLNLLDNTIMRT--DARYIIIVEKHAIFHRLVEDRVFNHIP-----------CVFITAKGYPDIATRFFLHRMSTTF------PDLPILVLVDWNPAGLAI(278)
Pp2   ADMNMKQHFYS----DARYILVIEKDAIFQRLVEERFFLKVP-----------CIIMTAKGFPDLASRALLHRLHQEF------PSMLIFALVDWNPAGLAI(177)
Aga   LDVGSVTEIRL----AAKLVLVVEKDTVFKRLLEDGISTFP---------DTLVLITAKGYPDVSTRLLLKKISDWT-------HVPIYGLMDADPHGIEI(173)
Am    HLASNVISARS----RARLVLVVEKDSVFQKLLEDDCTSSLN-----------CILVTGKGYPDVATRMLVKLLSEKL-------ELPVYIIVDADPFGIDI(296)
Ol2   GDLFELDATKIHS--DAAYVIVVEKDAVFNRLCAERVFEKLP-----------CVLVTAKGFPDLATRKFLHLLRRALEDNDRGNEAQFFGLVDWNPSGCWI(177)
Dm    TDPEKIDRIET----LAEFVLIVEKESVFESLLSRNVFGTFERR---------FILITGKGYPDCCTRRIVHRLTEEN-------QLAAYILVDADPFGVEI(229)
Dv    TDFENVERIVT----NAEMVLIVEKESVFESLLACNACNTIGLR---------FILLTGKGYPDCTTRRIVHRLSVEC-------NLPAYILVDADPFGIEI(247)
Ap    P-TPDLIEFLDV---DAEFVLVVEKDAVFQQLHRAGFWKKYK-----------ALLVTGSGQPDRATRRFVRRLHEEL-------KLPVYIITDSDPYGWYI(268)
Ss    P-TPDLIDFIDV---DAEFVLVVEKDAVFQQLHRAGFWKQYK-----------SILITSAGQPDRATRRFVRRLNEEL-------KLPVYILTDADPYGWYI(269)
Tn    V-NVDGLEIVKV---EASYVLVVEKDAIFQRLVREKFWSQES-----------AVLVTAKGMPDRATRRFIRRLNEEY-------KLPVYVLTDSDPYGWYI(257)
Hw    N-NPDTIDFLDH---DIDFVLCVETGGMRDRLIENGFDDMYN-----------CLIVHLKGQPARATRRITKRLHDEL-------SVPVVVFTDGDPWSYRI(257)
Dr    SNVNGIRNIVS----SAKFILIVEKDATFQRLLDDDFCARLSP----------CIIITGKGVPDVNSRLMVRKLWDAL-------HVPVFALVDADPHGIEI(272)
Xt    INVEGIRHLST----QAKFILIIEKDATFQRLLDDNFCARSGP----------CILITGKGVPDLNTRLLTRKLWDSF-------HIPIFTLVDADPYGTKL(285)
Mm    TNIQGMQHLIT----DAKFLLIVEKDATFQRLLDDNFCSRMSP----------CIMVTGKGVPDLNTRLLVKKLWDTF-------HIPVFTLVDADPYGIEI(285)
Nv    SHVDGIYDLYS----DAKVVLVVEKEATYHRLLDDGLLEKLNP----------CIMITGKGYPDVNTRQMVHRLWCTL-------HVPVMALVDADPHGIEI(294)
Nc    TLSASRYYTVSR---AGSGILVT---VRLCDLFCSNNADNY---------------TQGKGYPDLATRKFLAAIHSVR-------PRLLMFALVDFDPHGISV(292)
Pa    LPSTSEIRSIGFH--EVQWILLN---MQRTPEQVTESSSRYGFNITDDVFITKFSVCQAKGFPDLATRRFLSVVHSMR------PSLVIFGLADFDPDGISI(293)
OsD   LDQIKDVCVEIGSR-NIEYILVVEKHTMLNYLLEMDYHTNNN-----------CIILTGCGMPTLQTRDFLRFLKQRTG-------LPVFGLCDPDPEGISI(361)
Sp    ISHHRISSITS----TAKWVLVIEKEAVFQTLTEEALADT-------------IIVTAKGFPDLMTRKFLVKLAKALP------DAKFFGIFDWDPHGLCI(237)
Ago   SDSSCVSCKQWD---AVEAVIVVEKEAVFHRLTRAAAYCSNK-----------IIITGKGYPDQLTRVFLYRLLASAPR-----NVAVRAIADSDPYGIDI(267)
Sc    QDDAVIKLGTTS---MCN-IVIVEKDAVFTKLVNNYHKLSTN-----------TMLITGKGFPDFLTRLFLKKLEQYCS----KLISDCSIFTDADPYGISI(296)
Gz    VGSSYPNCSVNFWC-RHPRCQIDSGSGEGCRLQITLLLSILEGVS----------VWPRCTCHYLTTRSFLNLVSTRYP------QLPILGLFDFDPDGVKI(279)
Ca    YTKFFNTKITQIKV-EKVIVVILEKDAVFQCLCTHLRQHNIINR---------FLIVTAKGYSDNLTLRFLTWLQTN--E-----NCKFIGFFDSDVYGLNI(213)
Ps    HTKFEHILPNEE---SHYAIVILEKEAVFQSFCYYIKTRYTLKDNFVPDN---LIVVTGKGFPDNLTKKFVNILANTAFT-----NSVTLGFFDSDVYGINI(305)
Pp1   VHINKVSHLSS----SAEYILLVEKETVFQRLANDKFCAKNC-----------CILLTGKGYPDVATRSFLRRLVDQL-------HLPVYGLMDGDPYGLDI(180)
Gg    SNVQGIKNLYS----HAKFILIVEKDATFQRLLDDEFCIKLAP----------CIMITGRGIPDLNTRLLVRKLWDTL-------QIPIFTLMDADPHGVEI(297)
Sb1   VDIEAIKDVVS----VAHYILVVEKETVFQRLANDKFCERNR-----------CIVITGRGYPDIPTRRFLRYLVEQL-------HLPAYCLVDSDPYGFDI(271)
Hs    SNIQGIRNLVT----DAKFVLIVEKDATFQRLLDDNFCNKLSP----------CIMITGKGVPDLNTRLLVKKLWDTF-------HVPVFTLVDADPHGIEI(285)
Tg    SNVQGIKNLTS----HAKFILIVEKDATFQRLLDDNFFNKVSP----------CIMITGRGVPDLNTRLLVRKLWDSF-------QIPVFTLMDADPHGVEI(250)
Pt1   VHVEEVKDIVS----VANYILIVEKESVFQRLANDKFCNANR-----------CIVITGRGYPDIPTRRFLRLLVEKL-------CLPVYCLVDCDPYGFDI(246)
OsA   VSIEAIKDVVS----VADYILIVEKETVFQRLANDKFCERNR-----------CIVITGRGYPDIPTRRFLRYLVEQL-------HLPVYCLVDADPYGFDI(269)
Rc1   IHVEEVKDIVS----VAKYILVVEKESVFQRLANDWFCNAHR-----------CIVITGRGYPDIPTRRFLRLLLEKL-------GLPVYCLVDCDPYGFDI(249)
At1   VLVEEVEDIVS----LAEYILVVEKETVFQRLANDMFCKTNR-----------CIVITGRGYPDVSTRRFLRLLMEKL-------HLPVHCLVDCDPYGFEI(249)
                                                                                                        *
```
