# Supplementary material for: The double-stranded break-forming activity of plant SPO11s and a novel rice SPO11 revealed by a Drosophila bioassay
Source: BMC Mol Biol. 2012 Jan 16;13:1. doi: 10.1186/1471-2199-13-1 (PMC3273433; doi:10.1186/1471-2199-13-1)
Supplement: Additional file 2 — Supplementary Table S1: Primer sequences (5' to 3'). [file 1471-2199-13-1-S2.DOC]

## Additional file 2 – Supplementary table S1: Primer sequences (5’ to 3’).

| **Cloning of *OsSPO11* genes**  OsSPO11A 5’ region  mk-198 ACCATGGCGGGGAGGGAGAAGAGGCG  mk-196 CTCCAACCTCCATTGTGGGGCTTCCCTG  OsSPO11A 3’ region  mk-194 GCTGCATTGTGATTACAGGAAGAG  mk-208 TTATATATGTCTTCCTTGTTTGATC  OsSPO11B 5’ region  mk-190 CCCATGGCGGAGGCGGGAGTGGCGGC  mk-230 GCGTAGGAGAGGTACACCGACGAG  OsSPO11B 3’ region  mk-221 GACGTCTCGTCGGTGTACCTCTCC  mk-191 GGAATTCAAATGTAATCACCCTGTACAATC  OsSPO11C  mk-188 GGAATTCATGTCGGAGAAGAAGCGCCGCGG  mk-189 GGGATCCTCAAATCCAGTCCTGTTGCTGC  OsSPO11D 5’ region  mk-251 CCCATGGATGATTCAACGGATGACGATTC  mk-252 GCCTGTGTGGTATCTGCACTCCAATAG  OsSPO11D 3’ region  mk-192 CCCATGGAGTTTAATGAGGAAGTCAAG  mk-193 GGAATTCAGTCCTCACTGGCTTCAGTGTC |
| --- |
| **Subcloning into the pCasper-hsp83 vector**  AtSPO11-1-F-Ba TTTGGATCCATGGAGGGAAAATTCGCTA  AtSPO11-1-R-Kp TTTGGTACCTCAAGGAGAGCTTACTTCA  AtSPO11-2-F-Bg TTTAGATCTATGGAGGAAAGTTCAGGACT  AtSPO11-2-R-Kp TTTGGTACCTTATATGTATTTGCCTTGCA  OsSPO11A-F-Bg TTTAGATCTATGGCGGGGAGGGAGAAGAG  OsSPO11A-R-Kp TTTGGTACCTTATATATGTCTTCCTTGTT  OsSPO11B-F-Ba TTTGGATCCATGGCGGAGGCGGGAGTGGC  OsSPO11B-R-Kp TTTGGTACCTCAAATGTAATCACCCTGTA  OsSPO11D-F-Bg TTTAGATCTATGGATGATTCAACGGATGA  OsSPO11D-R-Kp TTTGGTACCATGGATGATTCAACGGATGAC  Each sequence contained a *Bam*HI, *Bgl*II or *Kpn*I restriction site (underlined). |
| **Quantitative real-time RT-PCR of OsSPO11 genes**  OsSPO11A-F GTTTGGTGGATGCAGACCCTTA  OsSPO11A-R ACCCCAAGCCACCGAATATC  OsSPO11B-F TCTCCTACGCCTCCTGCAA  OsSPO11B-R GCTTCCCCTCCCCCAAAA TC  OsSPO11C-F CTGCTTGAGGAGGACTTTGTGAAG  OsSPO11C-R TGGAAACCAAA TGAACTGAGAGCC  OsSPO11D-F GTTGGGTTGGATTGTCATCCTCAG  OsSPO11D-R TCGTCCTGCCAAAGGTTCTTCAAC  Actin-F-AK072796 CAAGGGCCGTGTTCCCTAGT  Actin-R-AK072796 CTTCTGCCCCATTCCTACCAT  AtSPO11-1RT-F GTCTTTTGCCACTGACAGAA  AtSPO11-1RT-R TCGATCTCAAACTTGACCCC  AtSPO11-2RT-F ACGTTGTAGTAGATACAGTCTTGG  AtSPO11-2RT-R TGTCTAGCAAATTCAAGTCTCC  DmSPO11RT-F TCCAAGGGCCTGGTGGCAG  DmSPO11RT-R AGCACAAATTCCGCCAGCG  RP49-F TCCGCCCAGCATACAGGC  RP49-R CAATCCTCGTTGGCACTCACC |
